# Molecular Epidemiology of Different Hepatitis C Genotypes in Serum and Peripheral Blood Mononuclear Cells in Jahrom City of Iran

**DOI:** 10.5812/hepatmon.16391

**Published:** 2014-05-11

**Authors:** Asghar Ashrafi Hafez, Rasoul Baharlou, Seyed Dawood Mousavi Nasab, Abbas Ahmadi Vasmehjani, Mohammad Shayestehpour, Negar Joharinia, Nayeb Ali Ahmadi

**Affiliations:** 1 Proteomics Research Center, Shahid Beheshti University of Medical Sciences, Tehran, IR Iran; 2 Department of Microbiology, Jahrom University of Medical Sciences, Jahrom, IR Iran; 3 Department of Virology, Faculty of Medical Sciences, Tarbiat Modares University, Tehran, IR Iran; 4 Department of Virology, Tehran University of Medical Sciences, Tehran, IR Iran; 5 Department of Medical Lab Technology and Proteomics Research Center, Faculty of Paramedical Sciences, Shahid Beheshti University of Medical Sciences, Tehran, IR Iran

**Keywords:** Hepatitis C Virus, Genotype, Polymerase Chain Reaction

## Abstract

**Background:**

The Hepatitis C Virus (HCV) is considered essentially hepatotropic, yet the virus compartments have also been found in important extra hepatic sites. Detection of HCV RNA in extra hepatic reservoirs such as peripheral blood mononuclear cells (PBMCs) is important for determining disease progression and treatment effectiveness.

**Objectives:**

The present study aimed to determine different HCV genotypes in patients' plasma and PBMC specimens, in Jahrom city of Iran.

**Patients and Methods:**

Blood samples of 137 patients with established HCV were collected at the Honari clinic. These patients were anti-HCV and plasma HCV RNA positive. After plasma RNA extraction and obtaining a pellet of approximately 3-5 × 10^6^ PBMCs, Real-time PCR was performed, using specific-genotype primers. Finally, data analysis was done by the Statistical Package for Social Sciences (SPSS) software.

**Results:**

Subtype 3 was the most common genotype in plasma (57.7%) and PBMCs (51.1%). Subtype 1a was detected in 36.5% and 30.7% of plasma samples and PBMCs, respectively whereas subtype 4 was not detected in any of the cases. There was a genotype difference between plasma and PBMCs of 12.4% of patients. In four patients no genotype was detected in their plasma but genotype 3 was detected in the PBMCs.

**Conclusions:**

It is suggested that determination of the target genotype by plasma subtyping for choosing the proper antiviral therapy is essential but may result in therapy failure. HCV genotyping in PBMC samples, along with plasma specimens, might be more beneficial. Therefore determining the HCV genotype in PBMCs, before beginning the therapy is useful due to the possibility of occult infection detection.

## 1. Background

Hepatitis C virus (HCV) belongs to genus Hepacivirus and the Flaviviridae family, and was first isolated in 1989 (1). HCV is a health problem, involving 170 million (approximately 3%) individuals around the world (2). HCV genomic material is composed of a positive-sense single stranded RNA of approximately 9600bp. After the primary infection, whichis often asymptomatic, a significant number of infected individuals, (60-88%), will develop chronic hepatitis C (CHC), which may gradually result in fibrosis, cirrhosis and hepatocellular carcinoma (HCC). In fact this virus is among important agents of HCC and cirrhosis (3, 4). The prevalence rate of HCV infection is 0.2-40% in different countries (about 0.2-2.2% in developed countries and near 7% in developing countries) (5-7). The presence of at least seven major types has been identified by comparison of nucleotide sequences of HCV strains, isolated from infected patients from different geographical areas (8). Over 65 different subtypes are distributed worldwide (9, 10). The HCV genotypes should certainly be considered before beginning the therapy, to determine the duration of the therapy, the amount of ribavirin and PEG-interferon administrated and as a strategy for monitoring virological responses (11). Hepatitis viruses such as HCV are considered as essentially hepatotropic (12, 13), yet HCV compartments have also been detected in major extra hepatic locations, including peripheral blood mononuclear cells (PBMCs), central nervous system (CNS) and bone marrow of chronically-infected individuals (14, 15). Persistence of the low amounts of replicating virus in PBMCs, even if HCV RNA is undetectable in patient's serum, may cause infection reactivation in the future, particularly if immune suppression takes place (long-term therapy, organ transplantations or HIV infection). The HCV sequences in PBMCs are different from those detected in plasma and liver samples (16, 17), which means that the virus genotype in serum may be different from PBMCs (e.g. 1a genotype in serum vs. 3a in PMBCs). In addition, differences in HCV quasispecies and genotypes have been detected in PBMCs, compared to the plasma, suggesting divergent evolution in these compartments (18, 19). Moreover, it may also play a role in the pathogenesis of extra hepatic manifestations of HCV infection (20). Detection of HCV RNA in extra hepatic locations has important impacts on illness progression, transmission and therapy effectiveness (21). Therefore, it is essential to determine the HCV genotype in PBMCs before beginning the therapy. The pathological significance and the relationship between HCV RNA serum levels and PBMCs is not well known in Iran and only one study has been conducted on this subject (22). This is due to the scanty and conflicting data available based on sustained virological responses (SVRs), obtained when HCV RNA is undetectable in PBMCs. Herein, there are no published data about the presence and prevalence of various HCV genotypes in plasma and PBMCs in Jahrom (South West of Iran) city, therefore the present study was conducted to investigate this issue in detail.

## 2. Objectives

The present study aimed to distinguish different hepatitis C genotypes in plasma and PBMCs, of patients from Jahrom city, Iran.

## 3. Patients and Methods

### 3.1. Study Population

In this cross-sectional study, 137 established anti-HCV positive individuals, referred to the Honari clinic (affiliated with the Jahrom University of Medical Sciences, Jahrom, Iran) from November 2012 to March 2013 were enrolled. Written consent forms, compiled by the Ethics Committee of Jahrom University of Medical Sciences were signed by the patients. Inclusion criteria were positive anti-HCV accompanied by the presence of plasma HCV RNA. Patients who were positive for anti-HCV antibodies but negative for HCV RNA were excluded from the study. Anti-HCV detection was performed by a third generation enzyme immunoassay, based on the manufacturers’ guidance (Diagnostic BioprobesSrl, Milano, Italy). Hepatitis C plasma RNA was detectedby thereal-time PCR method. Plasma samples were kept frozen and stored at -70°C until use. Also, a questionnaire, consisting of the demographic characteristics and risk factors, as the possible modes of transmission (intra-venous drug abuse, multi-transfusion and blood transfusion) was completed for each patient.

### 3.2. HCVRNA Extraction and cDNA Synthesis

According to the manufacturer's instructions, RNA was extracted from 100 µL of plasma, using the AccuPrep Viral RNA Extraction Kit (Bioneer, South Korea). About 5 mL of peripheral blood was kept in EDTA-containing tubes and then the PBMCs were isolated, using Ficoll-Hypaque density gradient centrifugation (Ficoll-Hypaque; Pharmacia, Uppsala, Sweden). The PBMCs were then washed three times with phosphate-buffered saline (pH = 7.3±0.1) and counted and then RNA was extracted from a pellet of approximately 3-5 × 106 PBMCs, using the RNA Virus Mini Extraction Kit (Invitek GmbH, Germany). The final volume, in the reaction for synthesis of cDNA, was 20 µL, including 5 µL RNA, 1 µL random hexamer (Fermentas GmbH, Germany), 6.5 µL of diethylpyrocarbonate (DEPC) treated water, 4 µL reverse transcriptase reaction buffer 5x, 2 µL dNTP, 10 mM stock, (Fermentase GmbH, Germany), 0.5 µL RNase Inhibitor (Fermentas GmbH, Germany) and 200 IU of Moloneymurine leukemia virus reverse transcriptase (Fermentas GmbH, Germany), which was then incubated at 65°C for 5 minutes, 25°C for 10 minutes, 42°C for 60 minutes and 70°C for 10 minutes.

### 3.3. HCV Genotyping

RNA was isolated from plasma and PBMC specimens as described above. HCV genotype was determined using type-speciﬁc primers and the protocol described previously (23). Briefly, genotyping was done using specific-genotype primers (1a, 1b, 2, 3, and 4) based on the amplified 5´-untranslated region (5´-UTR) of the HCV genome. Each type-specific HCV primer was 5´ fluorescently labeled. The primer sequences, the dye labels, and the sizes of the expected products are listed in Table 1. Firstly, reverse transcription (RT)-PCR with primers KY80 (5´-GCA GAA AGC GTC TAG CCA TGG CGT-3´) and KY78 (5´-CTC GCA AGC ACC CTA TCA GGC AGT-3´) to amplify a 244bp of the 5´-untranslated region (5´-UTR) of the HCV genome was performed. We used different samples with specific HCV genotypes as positive controls. Each 25 µl reaction mixture contained of 5 µl of template, 2.5 µl of 10X reaction buffer (Applied Biosystems, Foster City, CA), and 1.5 mM MgCl_2_ (AB), 0.5 mM of each dNTPs (AB), HCV-speciﬁc primers, and AmpliTaq DNA polymerase (AB). The amount of specific-HCV primers in each reaction based on the type of primer was different. Extension reactions consisted of initial denaturation at 94°C for 20 seconds, followed by 30 cycles of 94°C for 20 seconds, 64°C for 20 seconds, and 72°C for 35 seconds. The reaction was performed using 750 Real-time PCR (Applied Bio-system, USA). Genotyping was confirmed by sequencing with gold standard methods therefore we sent several samples for sequencing and results were compared with other sequences in GenBank by the GeneScan 3.1 software.

**Table 1.** Sequence of Primers Used for HCV Genotyping

Primers	Primer Sequence ^a^	Products Size, bp
**HCV - 1a**	5´ HEX- ACT CGG CTA GCA GTC TT 3´	193
**HCV-1b**	5´ FAM- ACT CGG CTA GCA GTC TC 3´	193
**HCV -2**	5´ FAM- TAT CCA AGA AAG GAC CCA 3´	137
**HCV -3**	5´ FAM- CAA CAC TAC TCG GCT AGT 3´	200
**HCV -4**	5´ HEX- CAT GGC GTT AGT ATG AGT GTT 3´	229

^a^ 5´fluorescent labels: HEX, 6-carboxy-2´, 4, 4´,5´,7,7´- hexachlorofluorescein; FAM, 6-carboxyfluorescein.

### 3.4. Statistical Analysis

Analysis was performed using the Statistical Package for Social Sciences (SPSS) software version 17.0, using descriptive values such as mean, standard deviation and Fisher’s exact probability test. P value < 0.05 was considered statistically significant.

## 4. Results

One hundred and thirty seven patients with positive chronic HCV were enrolled in the present study. Injection drug being the major risk factor (in injection drug users (IDUs)) was detected in 94 (71.5%) patients, blood transfusion was found in 23 (16.7%) patients and the cause of infection was unclear in 20 (14.5%) patients. The mean age of patients was 41.3 ± 11.6 years (range: 21-68). Of the 137 patients, 113 (82.5%) were male. The most common genotype in the study population was subtype 3 for plasma (57.7%) and PBMCs (51.1%), followed by subtype 1a in plasma (36.5%) and PBMCs (30.7%). Genotype 2 and 1b were mixed with genotype 1a and 3a whereas genotype 4 was not detected in samples. Subtype 3 was the most frequent genotype in patients over 40 years of age (47.3% versus 42.4%) and subtype 3 was the most frequent in patients under 40 years old (46.5% versus 34.9%). subtype 3 was more frequent (42.8%) in male cases. It should also be noted that the difference in the distribution of the genotypes between males and females was statistically significant (P value = 0.001). Also the most common genotype in HCV in IDUs population was subtype 3, which was more significant than the other subtypes (P value = 0.003). HCV types in plasma and PBMCs of all patients were analogous except for 17 patients (12.4%). In four patients no genotype was detected in the plasma but genotype 3 was detected in PBMCs. Detailed information on these patients is shown in Table 2. More than one HCV genotype was found in some patients; 5 (3.6%) patients had different HCV genotypes in their plasma samples and in 10 (7.3%) patients, mixed infection were detected in extra hepatic sites such as PBMCs. Mixed genotypes of 1a and 3 in PBMCs were the most common (As shown in Table 3). The genotyping by Real-Time PCR was approved by sequencing of the 5´-UTR. A complete correlation was demonstrated between HCV genotyping and sequencing of the 5´-UTR.

**Table 2.** Distribution of Different HCV Genotypes in Plasma, PBMCs ^a^

No.	Gender/Age, y	Risk Factors of Transmission	HCV Genotypesin Plasma	HCV Genotypesin PBMCs
**1**	Male/42	IDUs	1a	3
**2**	Male/47	IDUs	2/3	3
**3**	Male/36	IDUs	1a	1a/3
**4**	Male/53	IDUs	3/1a	1a
**5**	Male/43	Unknown	3	2/3
**6**	Female/48	IDUs	3	1a
**7**	Male/51	IDUs	3/2	1a/3
**8**	Male/48	IDUs	3	1a
**9**	Male/47	IDUs	1a	1a/3
**10**	Male/34	IDUs	1a	1a/1b
**11**	Male/43	Unknown	1a	3/1a
**12**	Male/48	IDUs	1a	1a/1b
**13**	Female/41	IDUs	2/3	3
**14**	Female/42	IDUs	1a	2/1a
**15**	Male/26	IDUs	1a/1b	1a
**16**	Male/52	IDUs	1a	1a/3
**17**	Male/48	Unknown	3	1a/3

^a^ Abbreviations: HCV, Hepatitis C virus; PBMCs, Peripheral Blood Mononuclear Cells.

**Table 3.** Frequency of HCV Genotypes in Plasma, PBMCs, and Liver Biopsy Specimens ^a,b^

-	HCV Genotypesin Plasma	HCV Genotypesin PBMCs
**HCV genotypes**	-	-
**1a**	50 (51.1)	42 (30.7)
**3**	79 (57.7)	50 (36.5)
**Mixed infection **	5 (3.6)	10 (7.3)
**1a/2**	0 (0.0)	1 (0.7)
**1a/1b**	1 (0.7)	2 (1.4)
**3/1a**	1 (0.7)	6 (4.4)
**3/2**	3(2.1)	1 (0.7)
**3/1b**	0 (0.0)	0 (0.0)

^a^ Data presented are No. (%).

^b^ Abbreviations: HCV, Hepatitis C Virus; PBMCs, Peripheral Blood Mononuclear Cells.

## 5. Discussion

This study was done on 137 infected chronic patients to trace the frequency of HCV infection in their plasma and PBMCs. In former studies there has been evidence of persisting HCV RNA in PBMCs despite sustained clearance of HCV RNA from serum (24, 25). Therefore, detection of HCV RNA in PBMCs is essential. Of course, tracing of HCV RNA in extra hepatic sites other than plasma, such as PBMCs is important for prediction of response to antiviral therapy (26). In our study, HCV RNA sequences were not detected in four plasma samples but were found in all PBMCs samples. In 12.4% of these patients various HCV genotypes were detected in, plasma and PBMCs samples. Several HCV genotypes were detected in 5 (3.7%) of the 133 plasma and 10 (7.3%) of the 137 PBMCs samples. The data presented here are in accordance with previous studies (22). Mixed infection is infection of a patient with two or more HCV genotypes. Mixed viral infections are clinically significant and may lead to more progressive illness, unresponsiveness to antiviral treatment or relapse after the end of the antiviral therapy period and persisting detection of HCV RNA in PBMCs or other extra hepatic sites (24, 27). In the present study, HCV subtypes presented in PBMCs varied from those detected in the plasma (Table 1). This means that PBMCs may present different subtypes, which are not found in plasma specimens therefore HCV RNA in PBMCs may indicate persisting infection, which result viral sustained implications. Furthermore, our findings confirm former proposed approaches in which PBMCs have been accounted as an extra hepatic replication location for HCV (12, 20, 28, 29). Therefore it is suggested that infection with one HCV subtype doesn’t block attainment of other genotypes, so several exposures to HCV, especially for risk groups such as IDUs and blood transfusion patients, might result multiple events of reinfection and mixed infection in patients. It is also well determined that super infection with a new HCV type causes prevention of another type under the detection limit of PCR while the other kind dominates under antiviral treatment; the alternate type may lead to viremica again and shift the result of the treatment (27, 30, 31). In addition, occult HCV infection has been characterized in the liver of anti-HCV and serum HCV RNA negative patients and it has been reported that viral RNA could be present in the PBMCs of nearly 70% of these patients despite undetectable genomic HCV RNA and antibodies against HCV in the plasma (32, 33). An important point of this study was that no HCV RNA was detected in four plasma patients while presence was found in PBMCs. Therefore, these patients had occult HCV infection. The HCV genotyping of HCV RNA detected in PBMCs of individuals with occult HCV infection showed that all of the patients were infected with HCV subtype 3a; this finding is similar to previous reports (34-36). Despite the fact that occult HCV infection has been recently identified, it has been reported from various regions of the world for example; it was seen in individuals with cryptogenic liver disease in Egypt (10%) (37) and Iran (10.1%) (36), individuals with cryptogenic cirrhosis with HCC in Italy (40%) (38), patients with lymphoproliferative disorders in Iran (1.9%) (36), and in patients with active hepatitis B (HBV) infection (28%) in Italy (39). However, there are also several studies in which scientists have not been able to trace the occult HCV infection, such as in mixed cryoglobulinemia; thus it is essential to perform more studies with large number of subjects, in this field (occult HCV infection) on different groups such as IDUs and high-risk groups.

However since liver biopsy is invasive, therefore all of patients must provide informed consents. Furthermore, we postulated which of these cases had occult HCV and determined that the therapy strategy is important. These patients had HCV RNA in PBMCs persistence, which may lead to HCV reactivation or further relapse after stopped interferon treatment at 24-48 weeks and under special conditions such as immunosuppression. These individuals require more monitoring after the end of treatment of antiviral therapy. Herein tracing of HCV RNA in the plasma before therapy is sufficient and PMBCs specimens can provide supplemental information when considering the therapy strategy. In our study the frequency of mixed HCV infection was determined as approximately 3.7% in plasma and 7.3% in PBMC these results are similar to other study from Iran (22). Mixed infection with two HCV genotypes has been found in 1% of HCV-positive patients, using type-specific primers (40); also 1.6% to 31% have been accounted by multi-transfused hemophiliacs (41, 42) but mixed infection in IDUs has not been reported in Iran. Similar to our results, a limited number of studies have reported that the 3a genotype in these individuals is common (43). This study showed that in HCV infected individuals, there is a significant relationship between having various HCV types and presence of genotypes in PBMCs specimens that are not found in the plasma, this is similar to other study (22). In this study we used specific-primer genotyping which has more sensitivity than restriction fragment length polymorphism (RFLP) assay and INNO-LiPA™ HCV genotyping. Therefore, the actual ratio of mixed infection may have not been minimized as found with the two other methods (44, 45). The main failure was that no patients had provided consents to undergo a liver biopsy (as hepatocytes are the main reservoirs for HCV). Since the usage of liver biopsy was not possible for the patients, we considered the evaluation of PBMCs as another important HCV reservoir for detection of HCV mixed infection before starting the therapy (26, 32, 33). In conclusion, this study showed that patients with IDUs are the most high-risk group in whom mixed infection is relatively common while failure of therapy and reversion of infection is frequent in this group. Thus, it seems that considering the plasma genotype, as the target genotype for determination of antiviral therapy, may lead to the failure of treatment. The determination of HCV genotype in PBMCs is beneficial for detecting HCV occult infections.
